# Editorial and Miscellaneous

**Published:** 1861-04

**Authors:** 


### EDITORIAL.

ERRATUM.—Upon page 144 heading in our March number, for PHYSIOLOGISM read PHYSIOLOGY. There are already *isms* enough in the world, without adding this barbar*ism*.

*Longview Lunatic Asylum. Report for* 1860. *Ohio.*—The building occupied by this institution is an elegant and spacious one, erected at a cost of nearly half a million of dollars. It accommodates about 400 patients, and is evidently conducted with great skill, sagacity and fidelity by its estimable board of officers.

*Dr. C. A. Logan on Miasmatic Fever. Leavenworth, Kansas,* 1861.—A monograph devoted to argument against the very convenient, but unsubstantiated, malarial hypothesis, and to show that the true cause of the so-called Miasmatic Fever is to be found in the poisonous influence upon the nervous system of an undue accumulation of carbonaceous material in the blood, however produced. The importance of the functional activity of the liver and the spleen—the necessity of proper oxygenation of the blood, the influence of diet, &c., are severally dwelt upon with considerable force. We are obliged to the author for collecting many facts which tend to elucidate this obscure subject.

*The Movement Cure.* By Chas. F. Taylor, M. D. Lindsay & Blackiston, 1861.—This little work may for aught we know be made, or is already, the basis of some exclusive plan of treatment, which we sincerely deprecate; but, so far as can be judged from a cursory examination, is worth perusal.

It is freely illustrated with diagrams, explaining the particular movements necessary to call into exercise the muscles of each part of the body, and contains many suggestions likely to be of advantage in a systematic course of hygienic treatment.

---

*D. D. Slade on Diphtheria.* The Fiske Fund Prize Essay. Blanchard & Lea, 1861; pp. 84.—The reputation of Dr. Slade would warrant circulation to a work of much inferior general interest to the present. The essay embraces the general and special history of Diphtherite, its nature, causes, symptoms, progress, pathological anatomy and treament. It is as concise as is consistent with a clear elucidation of the subject, practical, and we believe will attain a degree of popularity, as it certainly deserves, altogether unusual for "Prize Essays."

---

*Treatise on Fever.* By Robert D. Lyons, K. C. C., late Pathologist in Chief to the British Army in the Crimea, &c., &c. Blanchard & Lea, 1861; 800. pp. 362.—The late period at which this work was received will prevent more than a brief notice in this number. A rapid survey of its pages convinces that it is an exceedingly valuable work. It evidently brings up all the knowledge thus far accumulated on the subject of Fever, and condenses and concentrates the whole into a moderately sized, but capital, volume. Dr. Lyons is evidently no hobby rider, but has brought keen perception, extensive acquirement, a large comprehensiveness of view and a pleasing style into full and vigorous exhibition in this work. In a subsequent number we shall not scruple to make copious extracts frem this really fascinating volume, and meanwhile advise you, Beloved Reader, to go to Keen's, or elsewhere, and buy, read and ponder.

---

*The Institutes of Medicine.* By Martyn Paine, A. M., M. D., L. L. D., Prof. of the Institutes of Medicine and Materia Medica in the Univ. of the City of New York; Corresponding Member of, &c., &c., with a portrait of the author. 800 pp., 1108 with 6 pp. of certificates appended. Sixth Edition. Harper & Brothers, 1860.—This massive volume is but one of a series of ponderous productions, to the aggregation of which the author has devoted the energies of his life. His name is familiar to the profession as the comparatively isolated expounder and defender of solidism, or vitalism, "pure and simple." In extent of reading, and industry of annotating and writing; in entire absorption of all his faculties into the service of the one fixed idea of his life; in persistent and unchangeable devotion to the building up of leaf after leaf of his immense volumes; in steady and constant search for all the particulars, however minute, which may by any mental device be made to sustain the theories which he seeks to sustain, Prof. Paine is rather German than American, a mediæval schoolman than one of the degenerate scions of this later and "faster" age. It is not a little amusing to notice that about all the various notices and commendations which the work of this author have received, have a reticence about them which does not commit the writers to an endorsement of his opinions. Their greatest eulogy consists in congratulating the profession on the fact, that there is one writer who will insist on keeping the several facts (among the mass of mere assumptions), upon which dogmatic vitalism is based, constantly before the professional mind. They give credit to Dr. Paine for casting an anchor to the windward, so that the ship may not go to pieces on the rocks of chemical or mechanical materialism. Perhaps they forget that, in this age of the world, voyages are not accomplished by vessels perpetually anchored. The philosophy of the author is an absolute *estoppel* upon all progress and discovery. He refers all the phenomena observable in the human body, to certain abstract properties; in his own language, "substituted for the Creator," "analogous to the soul," and "capable of annihilation." He denies the propriety of making use of our knowledge of the physical laws, of any sort, to explain any of the phenomena of health or disease. He has erected his mammoth volumes, "the principles laid down in these Institutes," as pillars of Hercules inscribed *ne plus ultra.* We have read "these Institutes" from title page to finis, carefully, and we know whereof we affirm when we say that there is not a single idea in the work which has not been antedated for centuries. In this statement, however, we must be understood as excepting the author's misrepresentations and malignant attacks upon particular individuals—in this respect he is eminently original. He never condescends to notice the utter overthrow of his positions by contemporary writers, he recognizes no such abstraction as "the good faith of authorship." Let the positive proofs that his castellated positions are but remediless ruins around him, be ever so evident, he coolly congratulates himself and the world that his "solitary position is becoming relieved." Glorying in his angular, crablike, or rather circular progression, as the locomotive of modern discovery dashes by him he apostrophizes it like a scolding Hecate: "A ha!—my fine fellow, you'll run off from the track one of these days; you had better adhere to the crawfish methods—or better still, to the cuttlefish! Lo, here are "these Institutes," my own cuttlefish obscurity!"

One word about the peculiar method by which the author carries on his argument. Wriggling along through a mazy labyrinth of propositions, when the connective ones ought to be established, when the single fact needed to cement the others is sought for by the reader, and necessitated by the exigencies of the system—*that fact is never developed*, the author refers you to some score of paragraphs where he assumes he has established it. Turn now to each of these references, and you shall find that, under each paragraph, he but refers you to others, *including those first read.* Logically speaking, he undertakes to sustain all his doubtful and untenable propositions by the "Fallacy of References."

Briefly the "Institutes of Medicine" is a work characterized by learning rather than wisdom; by subtilty rather than profundity, by verbiage rather than ideas. It is not to the careful reader an obscure work. On the contrary by ordinary industry of analysis, there is developed a transparency of its notions, which will positively prevent their general reception among thinking men. The immense pile erected with so much labor, converges to a pyramidal apex, upon which nothing can find foothold or support.

It is a professional Ptolemaic system, wandering helplessly among the Copernicans and Newtonians of modern times. We indignantly protest against this Babel tower, being in any sense confounded with or mistaken for the true Temple of Medical Science. Such books as these, and the horrible methods of practice based upon them, have done more to bring what is called "legitimate medicine" into contempt, and to foster Homœopathy and all its kindred systems of knavery and imposture into public favor, than all other causes combined. We can scarcely be astonished to find quacks of every hue quoting "these Institutes" to sustain their dogmas, as glibly as Mahometans quote the Koran. We conclude by saying this is one of those works which illustrates the proverb "A great book is a great mischief." It has the portrait of the author (stamping it as "*genuine*,") at the head, and the certificates of the press at its tail. Price $4.00—more or less.

---

*Gelsemin in Spermatorrhoea.*—The Am. Jour. Mat. Med. commends the following "each night on retiring:

℞ Gelsemin gr. ss. Lupulin gr. iij. M. Gradually diminish the dose as the patient shows signs of improvement. We have cured several severe cases with from six to ten doses."

---

*Chlorate of Potash.*—This article has very nearly upset all the old hobbies. One of our friends uses it even when the patient is moribund "to oxygenate the blood." Another, as a substitute for Calomel as "an antiplastic." Another, as a "recondite alterative." Another, as "a stimulant to the mucus membranes." Another, as "a refrigerant saline." Another, as an "expectorant of specific characteristics."

Another has found it an "arterial sedative;" and another cures consumption and perhaps corns with it. Crystalized Elixir of Life!

Seriously, we apprehend that the inordinate and indiscriminate use of this article is becoming a little too dangerous to continue merely ludicrous. It would seem to possess about as active properties as the Nitrate, but tending to elimination rather by the mucus membranes than by the kidneys. Aside from slight stimulant effects upon this membrane, we feel warranted in saying that there is no real evidence of its medicinal efficiency. It seems especially useful in certain morbid conditions of the respiratory mucus membrane. Employed, as it frequently is, in aqueous solution acidulated by Hydrochloric acid, it of course has to divide the honor of success with the acid of the menstruum. We really hope that this very clever little medicine may not be "run into the ground" by its present enthusiastic admirers. We have too much regard for it, in some cases and in small doses, as a very convenient *placebo*—cheaper even than Iod. Potassium.

---

*Infibulation.*—In these days of moral degeneracy we are sometimes led to look back with longing eyes upon "the good old times," when the world had not become practiced in iniquity, and young people were patterns of moral excellence. We are not disposed to sermonize, but in looking over an old edition of Fabricus Ab Aquapendente we find in CAP. LXIII, *De Chirurgicis Operationibus*, the *Ratio Infibulandi Adolescentes*—which, at the present, when Anti-Tobacco seems to be the only escape valve of hygienic enthusiasm, would appear to offer a new field of philanthropic effort. We roughly translate it from the original black-letter Latin—brushing away the dust of antiquity, we commend it to the consideration of medico-moral refomers.

"The infibulation of boys, according to Celsus, is accomplished in this wise:

It is performed sometimes on account of the voice, and sometimes on account of health, and the method is to extend the foreskin over the glans and mark upon each side the proper place for perforation, and then it is allowed to slip back again. If the marks are returned over the glans, too much is taken up, and it should be marked lower; if the glans is free from them, the proper place for the fistulæ is secured. Then the skin is transfixed through the marks by a needle carrying thread; the ends of this thread are tied together, and it is daily moved until the foramina are cicatrized; when this is perfected the thread is removed and the *fibula* is inserted. But this, indeed, says Celsus, is often greater than necessary. By this method of infibulation if the fibula should not be seen, nothing can be perceived, as Celsus remarks. Wherefore I am in the habit of exhibiting to my hearers an antique fibula, procured from the museum of the illustrious Pinellus, and apply it to the organ that they may see how youths may be preserved from sexual connection."

All are familiar, of course, with the methods of an analogous kind, formerly, and even now in certain parts of the world, adopted to secure the chastity of their females. Would it not enlarge the area of professional service to the world, if some of the gentlemen now howling themselves hoarse, and writing themselves blind, over the Whiskey and Tobacco hobbies, would—

"Leave off their damnable faces and begin"

to revive these ancient methods of moral reform, or rather prevention, of which we are told an ounce is worth a pound of cure?

---

*Influenza.*—This common and depressing malady, a correspondent of the New Orleans *Journal* observes, is no longer treated in England with purgatives and salines. Mulled wine is the favorite and successful substitute. One prescription used with much success is the following:

℞: Ammon. Sesquicarb. . . . . . . . . . . . . . . ʒ ss;

Ætheris Chlor . . . . . . . . . . . . . . . . . . . . ʒ j;

Tinct. Cardam. Comp. . . . . . . . . . . . . ʒ j;

Mist. Camph. ad. . . . . . . . . . . . . . . . . ℥ vj;

Ft. mist. partem quartam 4 tis horis.

*She-Doctors.*—Dr. Bennet Dowler is out in his editorial columns, in favor of recognition of a feminine branch of the profession. He advises consultation with them, provided they are properly educated and qualified. "Is not," says he, "the rule forbidding consultation with all females, regardless of their qualifications and merits, an unnecessary restriction? an aggression? an injustice which will react injuriously upon the profession, and give rise to well-grounded charges of prejudice and persecution?" Whilst we cannot but admire the chivalrous gallantry of our Southren *confrere*, we must be allowed to dissent totally from his conclusions. We do not propose to discuss the Woman's Rights question—all the world knows the dear creatures have the right to make doctresses, or worse, of themselves in "these United States," but another question arises when we coolly contemplate such a recognition of them, as to encourage others to fly off in such a deplorable tangent from their true orbit. We like women well enough, as an abstract proposition, but from the tongue of the sex in consultation, (*Horresco referens!*) we can only pray—"Good Lord deliver us!" Just think of this new apple of discord, tossed into the already not over peaceable professional circle! Would not the French critic's description of "the brilliant discourses which adorn the French Academy," be re-enacted every time He and She met over an unhappy patient? *Sic*—"A strife of words kept up by a confusion of principles; entire absence of conviction in one party, and extreme narrowness of views in another; fighting in empty space; reasonings in a circle; and false conclusions."

---

*Solutions of Iodine.*—From many recent experiments, the opinion seems to be gaining ground that, in order to develop its full powers as a local application, Iodine requires to be used in much stronger solutions than have usually been employed. Whilst Nitrate of Silver has been used in very strong or even saturated solutions, Iodine, perhaps from apprehension of its more highly irritating character, has been applied only in solutions of comparatively trivial strength. As an application to the Diphtheritic throat, to the cavity of abscesses, even into the serous cavities affected by disease, &c., &c., there are evidences accumulating that solutions of maximum strength are proving vastly more efficient, and freer from unpleasant consequences, than has heretofore been considered either probable or possible.

---

*Hypodermic Injections.*—Dr. Thayer, in the *Berkshire Med. Journal*, chronicles quite a number of cases of neuralgia and local pain from other causes, a case of dysentery, one of asthma, and one of delirium very promptly relieved by subcutaneous injections of solution of morphia—using generally about the sixth of a grain in q. s. of the menstruum.

---

*Bandages in Rheumatism.*—The *Berkshire Journal* strongly recommends bandaging of the affected limb as "a valuable adjuvant" in the treatment of Rheumatism.

---

*Bloodletting, et alii.*—Prof. Gross recently read a paper before the Philadelphia County Med. Society, wherein he enters an earnest protest against many of the medical ideas of the present time. The initial proposition is sufficiently significant of what is to follow. He says:

"The revolutions in medicine have been almost as numerous as the revolutions of empires, and hardly, if indeed any, less disastrous to human life. The checkered arch which spans its horizon, has everywhere inscribed upon it, in legible characters, the words change, change, change. Every age has had its medical absurdities and inconsistencies, and, judging from the tendencies of our own, it is evident the times are sadly out of joint in these particulars."

To stay this flood tide of change appears to be the great object of the author. He believes in the unchanging type of diseases; he believes in inflammation as at the bottom of all organic diseases; he believes in bloodletting as the especial remedy for inflammation, and he believes that the modern substitution of opiates, stimulants and sedatives, for the good old-fashioned lancet, is fraught with multitudinous danger. He quotes the case of the late eminent Dr. Francis, who once in a violent attact of croup lost, in a few days, nearly three hundred ounces of blood, and yet recovered. [It is a saddening commentary on this temporary escape of Dr. Francis, that his son, a young man of brilliant promise, was unquestionably hurried to his grave by an attack of Typhoid Fever, which was treated heroically by Bloodletting and Mercurials!]

He denounces Todd and John Brown, (not him of Harper's Ferry, but "the other man"), sneers at Virchow, cannot endure Bennett, and begs Young Physic to recollect that Hippocrates is looking down upon him from the height of nearly twenty-five centuries.

It seems a mere platitude to repeat "antiquity is but the youth of the world," but Prof. Gross appears to sturdily believe (with "the venerable Dr. Paine,") that what was taught by the magnates of mediæval or ancient times, is as rightfully binding upon the belief of the present day, as the experienced teachings of an old man upon a youth.

Now, with the highest estimation for the professional learning of Prof. Gross, we must say that, here and elsewhere, we believe he is misled by the adoption of utterly untenable premises.

1. He assumes that there has been no change in the type of diseases,—hence old time experience in their treatment and the evidence of the fathers, in favor of "depletory" and "antiphlogistic" methods of treatment, are to be received unhesitatingly.

2. He assumes, in fact although not in express terms, that the great variety of changes collectively denominated "inflammation," are essentially the same process, and hence amendable to the same treatment.

On one page he insists: "*There is no doubt that an early spoliative bleeding and the judicious use of the more heroic*

*articles of the materia medica, formerly so much in vogue, will often do more in saving structure and function than any other of the popular remedies of the day.*" (The italics are ours.) But upon the next page, apparently conscious of the dangerous application of this suggestion, in a description of his ideal of an "older and wiser" practitioner, he says that "*He administers medicine with a sparing hand, carefully eschewing everything that savors of polypharmacy and heroic measures.*" Is this a "sop to (the professional) Cerberus?" We are content to aspire to belong to Dr. Gross' "older and wiser" party.

The fact is "Brunonianism and Toddism," between which the parallel is attempted to be drawn, have no scientific feature in common. It is a logical error to assume that because in some respects things are similar, that, therefore, they are essentially alike. Scientific modern practice, as largely influenced by Drs. Todd and Bennett, is not to be put down or belittled by coupling it with "Brunonianism and other isms." The "reaction," or rather retrogression, so earnestly desired by Prof. Gross, is not to be secured by falsely supposed damaging comparisons, any more than the scientific physiology and pathology of the day is to be overwhelmed by the ponderous treatises which Prof. Paine has rolled up, by constant iteration and reiteration of the foggy lucubrations of Stahl, Van Helmot and the musty scholiasts of the dark ages.

---

*Ligation of the Arteria Innominata.*—Dr. E. S. Cooper's case proved fatal on the forty-first day, by secondary haemorrhage. Haemorrhage occurred once or twice to a slight extent but was controlled. Ultimately the patient, in a fit of either frenzy or despondency, tore off the dressings and speedily succumbed.

---

*Prof. Lawson* returns to the practice of his profession at Cincinnati this spring.

*Diphtheria.*—A correspondent in Indiana writes, in a postscript to a business letter, as follows:

"Diphtheria prevailed in this locality to a very considerable extent during the previous autumn. From actual experience and demonstration, I am prepared to prove that no benefit will accrue from the use of Nitrate of Silver as a caustic. I treated fifty-one cases without cauterization of any kind, and lost but two. I depend for the most part upon the constitutional treatment. I used Quinine and Chlorate of Potash freely—Calomel when I deemed it necessary. I have given to a child from three to four years of age, from 100 to 120 grs. of the Chlorate in 24 hours."

We hope our friend will communicate more in detail.

---

*Camman's Stethescope.*—This formidable looking toy can be obtained in this city, at or about $7.00, we believe. We have one in the office which hung up in sight for a year or two, but having accidentally ascertained that by some of our patients it was mistaken for a pair of obstetrical forceps, we incontinently stowed it away in the recesses of our deepest drawer. *Hic jacet!* It is a capital invention for magnifying the mistakes to which the common single-barrelled Stethescope gives rise.

---

*To Correspondents.*—We cannot undertake to reply by letter to all letters received, unless there is special occasion. Our list of receipts, it will be seen, cannot include those of a later date than ten days or a fortnight prior to the issue of the No. which contains the list. Where it is appropriate we shall respond through the Journal. We have promptly mailed all such back No's as have been applied for. To several parties we have mailed back No's twice, on information that those previously sent had not come to hand. If they have not been received it is not our fault, but must be charged upon the mails.

*Bills.*—In the present No. we enclose a statement of the accounts of all subscribers in arrears, as they appear on the books of the JOURNAL. If any errors are manifest, they will be corrected with great pleasure. It is positively incumbent upon us to collect in these bills. Pay a part if not the whole, but pay the whole if possible. There are not less than Three Thousand Dollars standing upon our books, even after excluding all those not believed to be against "good men and true." Meanwhile we are paying the printer from our scanty private funds. It is to be hoped there will be no more delay in settlement.

---

SINCE the re-organization of the Faculty of Rush Medical College, it has been a matter of constant concern and effort to enlarge and improve the Museum. Already the evidence of their united effort is visible in the greatly increased means of illustration, and rapidly growing collection. Valuable importations have been made from Europe in the different departments, besides the addition of very many useful and elegant specimens of home preparation.

New cases have been placed in the Museum the past year which are quite filled, and others will be added the coming summer, which it is the intention shall not long remain empty.

A few years will find the College in possession of a collection commensurate with its rapidly growing wants, and proportioned to the importance of its position.

The efforts of the Faculty can be materially aided by the Alumni and friends of the Institution having abnormal or other specimens in their possession, by a donation to, or deposit of them, in the College Museum. They are comparatively valueless to the private practitioner, being seen by but few, whereas in a Museum, visited by hundreds every year, their worth is proportionally increased. It is in the hands of the Teacher of Medicine that abnormal specimens attain their true value.

*City Medical Library.*—There are in Chicago somewhere from 175 to 200 regular physicians. There are, of course, an innumerable throng of quacks and nostrum peddlers of every hue, from the elegant specimen who haunts the outgoing cars of the Central Rail Road with his "*two-shilin-botl-warnted-to-cure-ed-ache-toothache-nd-any-kind-of-pain-in-five-min'ts*," down or up to the ponderous individual who vends small pills in marble fronted houses on *the* Avenue. But these latter we pass over for the 175 of the regular order.

There are two or more Medical Societies in the city and county, each useful in its day and generation. These societies are sometimes disposed to look with disfavor upon such professional gentlemen as do not join their ranks as members. Nevertheless the truth remains, that but a moiety of the profession belong to either. We propose trial of another method of association, and that is, the establishment at some central point in the city of a Medical Library, suitable to the wants of 175 or 200 reading professional men. A Library of "books which are books,"—a collection which may prove as valuable to our profession as the Law Library in the Court House proves to the legal gentlemen of the city. Let the library room be the professional exchange where physicians shall meet, both formally and informally, and compare notes and exchange views. Where the amenities of professional intercourse may be cultivated, at the same time that minds are enriched with valuable information. A moderate fee for admission to its privileges at the outset, and perhaps annually; donations, which would be abundant if permanency was guaranteed, and a little vigorous effort in the right direction, would soon give us a Library which would be an honor to the profession and the city. The details are simple and the methods evident. What say you, Gentlemen, whose lives and voices are elevated continually in the advocacy of "elevation of the profession?" Which of you will immortalize himself by moving in the matter?

Death of Dr. Fountain, of Davenport, Iowa.—Dr. Fountain's death occurred on Friday afternoon last, after great and continued suffering for a week, which he had borne with unflinching Christian fortitude. For some time past he had been continuing, at the suggestion of the American Medical Association, his researches upon the properties of chlorate of potassa as a remedy in phthisis, taking the ground that the article when pure was almost entirely harmless in large doses. Under this conviction, he took upon several occasions doses of half an ounce, and on Friday at 10 A. M. he took one ounce, dissolved in a pint of water. No serious symptoms occurred through the day, except a profuse diuresis and discoloration of the superficial circulation, and he visited his patients as usual. Having eaten a hearty supper in the evening, he returned to his house, where he was shortly after seized with severe pain in the abdomen, and so greatly prostrated as to be unable for some time to call assistance, (being alone in the house, his wife being absent at the East.) He expected to die in this condition, but by a desperate effort succeeded finally in calling his neighbors, who sent for his partner, Dr. Adler. His symptoms were after a time partially relieved, but soon he was seized with vomiting, ejecting a dark-colored, greenish fluid, being unable to retain any nourishment; the secretion of the kidneys was also entirely suppressed. This condition continued, with a gradually increasing prostration of the system, (the mind being perfectly clear,) for seven days.

"The post mortem examination revealed extensive inflammation and disorganization along the whole course of the intestinal canal, with adhesions agglutinating nearly the whole of the abdominal viscera; the gall bladder distended with a a thick dark colored fluid; the kidneys enlarged and lobulated externally, the internal surface and substance engorged, and the uriniferous tubes distended, containing frequent points of a crystalline substance, which was, without doubt, chlorate of potassa; and the bladder entirely empty, contracted and inflammed.

As a full statement of the case will be published, it is unnecessary for us to dwell further upon it.

In the death of Dr. Fountain the profession in the Northwest has lost one who was destined to prove one of its brightest ornaments, and the community in which he lived, one of its most valuable citizens.—*Chicago Tribune.*

We had little thought when penning the paragraph on

Chlorate of Potash, in another part of the present number of the *Journal*, that our ideas were so soon to have a striking and melancholy illustration. This sad experiment has shown that although the article in question is comparatively inert, in doses ordinarily employed, yet in inordinate amount it is capable of even fatal effects. In this respect it is altogether analogous to many other salines. We trust that this calamitous result may not fail to impress the needed lesson.

It is to be hoped that we shall hear no more of the intense folly of Chlorate of Potash being in any sense a remedy for phthisis. Phthisis never has been, and we hazard little in saying, never will be cured by medicine. If modern physiology and pathology have taught any one thing more than another, it is that this fearful disease is to be met by nutrients and appropriate regimen. Medicine therein is only indirectly and remotely beneficial. And yet we have known case after case go down to death, in this very city of Chicago, during the winter just past, treated empirically with Chlorate of Potash, expectorants and seclusion. Why, it is not yet a year since one of our most estimable physicians allowed himself to be treated for phthisical symptoms with small doses of antimonials and low diet! He fortunately escaped the immediate necessary effects, by seeking the natural tonic and stimulant effect of Lake Superior air and habits (including appropriate drinks) during the last summer, and during the winter the opportunities of out-door air and exercise, and consequent invigoration of the digestive system, afforded by a Southern climate.

It is our duty to make one other remark in this connection. Experiments made upon the healthy system, are of doubtful import, as illustrative of the effects upon the same system in a state of disease. The article which is rapidly eliminated by the healthy organ and thus proves harmless, may in a condition of disease prove deadly by its continuance in contact with the same. Experience demonstrates the fact, however it is explained.

Much confusion exists in some minds as to the terms medicines, poisons and food. Medicines and poisous are agents capable of producing a change in the structure or action of any part or the whole of the system, although constituting no part of the integral constituency. They are respectively poisons or medicines according as this change is not, or is, necessary. The degree of action does not constitute a scientific distinction in their nature. A medical action is never unnatural. It is a gross confusion of terms to call any medicine an unnatural agent. As well speak of the fire which restores the warmth of the body as an unnatural agent, because it can char and destroy the same body thrown into it. But food enters into the very structure of the body, and constitutes an integral part thereof. Not so entering or constituting, it is noxious, poisonous if you please, in kind if not in degree, as is woorara or arsenic. A like significance attends the idea of the influence of all the so-called imponderable agents. They are noxious or salutiferous according as the changes they tend to produce are not, or are, requisite.

With due regard to strictness in the use of language, there is not, and cannot be, a specific for any disease, phthisis or other. In this day it is as great folly to search for it, as for the "Philosopher's Stone" or the "Elixir of life" of old time imaginings.

Let the Homœopathists enjoy their specifics jointly with the other newspaper quacks,—we shall continue to steadfastly believe that, though hand join in hand, they shall not escape their destined punishment.

The very best remedy for a diseased organ, whether a tuberculous lung, an indolent ulcer or a gouty toe, is to wash it with healthy blood, flowing from within outwardly, and not by healing salves, inhalations, poultices and specifics, working from without inwardly, as the manner of some is.

The method of nature is to be imitated by art, but as Shakspeare observantly writes, "The art itself is nature." It is all very well to train the branches and graft them, to wash and peradventure whitewash the trunk of your tree, but if you wish it to grow and flourish and bear goodly fruit, you must supply the rootlets and spongioles with nutrient fluids, and surround the leaves with appropriate atmosphere.

One very considerably eminent gentleman is reported to have said that the great want of the present time is, a specific for tubercle, and a specific for cancer; as in Scabies we have Sulphur, and in Syphilis, Mercury, for specifics. The prayer of his heart can never be granted as he intended, although it may in fact. Because neither Sulphur nor Mercury are specifics within any correct definition of the term.

Specifics, whether Chlorate of Potash or Morrison's Pills, can have no existence anywhere, save in the imaginations of speculative theorists and hobby riders.

In these remarks we by no means would be thought to convey the idea, that the lamented Dr. Fountain was in any way committed to the idea that Chlorate of Potash would prove specific in Phthisis. We do not know whether he was or not; but we do know that many who had read his articles on the subject, have already mounted this hobby and were fast riding it to the death—of their patients. For Heaven's sake, my Chlorate-of-Potash-bitten friend, don't ride this poor hobby to the nether regions as quickly as you did Veratrum Viride, yesterday; Aconite, the day before, and Digitalis a little while since. Or were you straddling the Hypophosphites last week? We have medicines enough already—too many; would that we knew better how to use them.

Meanwhile, what do you say to a bit of beefsteak, tender, rare-done, washed down with generous beverages, (such as saved the threatened life of our friend above spoken of,) a little more pure air and sunlight, a clear conscience, sleep at nights and regular bowels? *These* afford a remedy for Phthisis worth thinking about.

---

*Ye Oliphaunt.*—It is whispered among the knowing ones of the city, that some of our professional brethren having drawn an elephant in a raffle, are in great straits what to do with him. We see countenances in which melancholy anticipations, scarce concealed, prey like a worm in the bud upon their *damaged* cheeks—What shall be done with "*ye oliphaunt?*"

Briefly then, as public journalists, we advise those implicated, —don't hang your lip or put finger in the eye, but draw your weasel-skins like men. That is "the long and the short of it," as a gentleman laboring under difficulties says when he compares his debt and credit pages.

---

Sir Benjamin Brodie's eyesight is pronounced lost beyond the hope of restoration. The London *Medical Circular* says:

"Prior to the late operation, the distinguished German oculist consulted in the case had expressed an opinion that the retina behind the cataract would be found to have retained its integrity; but a month has passed since the removal of the lens, and no improvement whatever has taken place. Objects of any kind are totally undistinguishable, and almost light from darkness. In other respects it is satisfactory to state that Sir Benjamiu is in good health, and bears his misfortune with becoming fortitude."

By other advices it is understood that the blindness is not so complete or hopeless as here intimated. Nevertheless the the worst is feared.

---

*Thesis.*—Dr. M. M. Eaton, of the late graduating class of Rush Med. College, furnished a very well written Thesis on Menorrhagia as his inaugural. It was inadvertently omitted from the copy furnished the printer last month.

---

*Marine Hospital at Detroit.*—The profession in Michigan are to be congratulated on the selection of Dr. Bliss, of Grand Rapids, as Surgeon of their Marine Hospital, by the new administration. Our bowels of compassion yearned over the imminent danger of the unfortunate sailors, contingent upon the appointment of another *strong* (in the olfactory sense) individual to the post. Dr. Bliss will honor both himself and the Government in the position.

*Homœopathy in the University of Michigan.*—It is rumored that the Legislature of Michigan have again attempted to foist one or more chairs of Homœopathy upon this institution. We opine the effort will fail as it has heretofore done. The Constitution of the State lodges the supreme control of the University in its duly elected Regents, and that body dare not shoulder the odium of any such folly. The only things homœopathic that are or will be permitted about the Ann Arbor College, are the fees for admission, and the brains of *two* of its ——perhaps that is going far enough among the infinitesimals.

---

*Chicago Marine Hospital.*—At the present writing, so far as heard from, the appointment of Surgeon to the Marine Hospital hangs fire. There is a rumor floating around the streets, however, that it is to be bestowed in a quarter which makes the doctors stare at each other in blank amazement and leaves them undecided whether to laugh or swear. As it is none of our funeral, we can only beseech the mourning disappointees to take another long breath, wipe their eyes and hurrah for "*Ol Dabe.*" Dear Brothers, there are *some* names with which treachery is a synonym. Which of you was cheated?

---

*The Cæsarean Section.*—A correspondent of an Eastern exchange, recently, coolly suggests that mothers with deformed pelves should be allowed in all cases to go their full time, and then trust to the chances of the Cæsarian section. It is suggested to our cotemporary who admits the communication, that that will do for *that* correspondent. *He has climbed high enough on that pole—take him down.*

---

*Ointment for Hemorrhoids.*—A good substitute for the Ung. Gallae is afforded by rubbing in a mortar from one to five grs of Acet. or Mur. Morphiæ with a little oil, and adding to this an ounce of lard or spermaceti ointment well mixed with from ten to thirty grains of Tannin. This is unirritating and efficient.

*American Medical Association.*—The Nashville *Medical Journal* makes the following timely observations:

"The founders of the American Medical Association were wise enough to foresee the metamorphosis of nations upon this continent, and in establishing a government for its profession of medicine, placed it above the reach of revolution. There is nothing *national* in the Association. It was designed for America with its one or one hundred nations. It is not the Medical Association of the United States—not at all. It is the American Medical Association. We have seen the profession of Canada represented in it, and hope to see members from Central and South America raising their voices in it for the general good. It could constitutionally hold its meetings in Vera Cruz, Montreal or Havana, as well as in Charleston or New York, and would do so upon invitation. We hope to see a large meeting at Chicago in June next. The Garden City can accommodate all that go. Let every physician of America, from ocean to ocean, and from the Gulf of Mexico to the North Pole, feel that

"No pent-up Utica bounds his view,"

and that he owes it to his profession to stir up the members of his local medical society to see to it that a full representation shall be had."

It is understood that the energetic and zealous Committee of Arrangements have secured Metropolitan Hall for the session of the Association. It is perhaps to be regretted that the better characteristics of the new Bryan Hall, in respect to acoustics, ventilation and beauty, had not commended it to the committee in preference. It may not be too late even yet to remedy this apparent mistake. Nevertheless we can assure our visiting friends that the quarters secured are equal, or superior, to any which have heretofore been placed at the disposal of the Association.

We fervently trust that Chicago shall prove in June next a true medical Mecca, whither the Æsculapian tribes shall come up and revive if possible the ancient glories of the profession.

---

*Extirpation of the entire Parotid.*—M. Marzolo relates a case, in the *Gazetta Medica Italiana*, wherein he extirpated the entire parotid gland, preserving the facial nerve and the external carotid artery. In six weeks the cure was complete; and eleven years after, the patient, a woman of fifty, had presented no indications of a return of the disease. The *Gazette Médicale de Paris*, which also records the case, appears not to be perceptibly astonished—although consternation and wrath may, perhaps, run riot in the classic purlieus of *L'Ecole de [illegible]atique.*

---

*Free Openings into Suppurating Joints.*—There is very decided progress in opinions, with reference to the propriety of freely opening synovial cavities, where evidences of suppuration are present. The danger of admission of air has been, clearly, over-estimated. The advocates of speedy opening have, recently, adduced powerful support of their position by published cases. Not only is immense pain and suffering spared the patient, but recovery with unimpaired function of the joint is manifestly promoted. Even when the larger serous cavities become involved in the suppurative process, it is beginning to be understood that not only may the contents be safely and advantageously discharged by operation, but their surfaces may often be hopefully treated by local medicinal applications. Germane to this proposition, we are promised the details of a case of pleural empyema, wherein a solution of Iodine, of almost heroic strength, was injected with the most admirable beneficial effect, including a speedy and permanent cure.

We opine that pure air is not so dangerous either to the internal or external parts of the body as some of the advocates of "seclusion in phthisis" and sundry other disorders seem to imagine. The advantages of freedom of discharge largely counterbalance all theoretical fancies about the disastrous effects of air.

*Epitome of Braithwaite's Retrospect.* By Walter S. Wells, M. D. New York: Chas. T. Evans. For sale by W. B. Keen & Co., Lake Street.—We chronicle the issue of this work with very great pleasure. In two volumes of a little over 900 pages each, we have collected the cream of this well known Retrospect. Having examined the volumes with care, guided somewhat by our own copious index of the original prepared with very great labor, we freely say that the Epitome is really more valuable than the complete serie All the subjects treated of are collated in alphabetical order, and crowned with a full index, so that any subject can be referred to at once. At a moment's glance you can find all of improvement, advance or innovation, that Braithwaite has gathered up in the last twenty odd years—a period richer in progress than any other known to history. The Editor has performed his part of the work with great industry and sagacity. We hesitate not in saying that no physician, who has any idea of "keeping posted," can afford to do without the Epitome. The advertisement, being a matter of permanent interest, can be found on another page.

We are gratified to notice that the very capable Editor is preparing a Summary of Medical Sciences to be issued by the same publishers semi-annually.

---

CHICAGO, April 3d, 1861.

ED. CHICAGO MEDICAL JOURNAL:

Since handing you the article to be contained in another part of your *Journal*, I have seen a communication from the pen of Prof. A. K. Gardner, appearing in the *American Medical Times*, wherein he claims priority of invention and asserts the superiority of his "Uterine Elevator" over that described by me in the *Times* a few weeks before.

I cheerfully concede the rights of the Professor, so far as they regard mere antecedence in the application of the mechanical principle involved, though, I may be permitted to add, I was not myself, nor were the professional gentlemen to whom I submitted my instrument and by whose advice I published the account of it, aware of the existence of his "Elevator." I had consulted the latest catalogue of Messrs. Tieman & Co., and found there a notice of *Sanger's*, *Sims & Elliott's* instruments, and had taken pains to see them, hoping to meet *in them* the requirements of a case then under treatment. No mention of Dr. Gardner's instrument is made in that catalogue, and my ignorance of his invention, therefore, needs no further apology.

As to the alleged inferiority of my "Retractor," a practical knowledge of its efficiency inclines me in no respect to defer to the opinion of the distinguished Professor. The axis of the instrument, when in position, is near enough the os uteri for all practical purposes, and in fact, by the distance of "half an inch" intervening, perfect security for the cervix is gained. In my opinion, the "Retractor" is not so obnoxious to criticism, on the score of liability to get out of order, as is the instrument of Dr. Gardner, the working parts being entirely enclosed, yet not beyond the reach of lubricating oils.

In conclusion, I still maintain the "Retractor," as neither too "clumsy and heavy," nor of less power than is perfectly sufficient for the object proposed in its construction.

Dr. Gardner's instrument must be used in but one direction, the stem being capable of motion, so far as I may judge from his diagrams, to the extent of three-quarters of a circle, nor is the operator able to detect the exact position of the uterus by means of any mechanical appliance, as is the case with the instrument devised by myself.

Yours very respectfully,

H. WEBSTER JONES, M.D.

